# KLF9 (Kruppel Like Factor 9) induced PFKFB3 (6-Phosphofructo-2-Kinase/Fructose-2, 6-Biphosphatase 3) downregulation inhibits the proliferation, metastasis and aerobic glycolysis of cutaneous squamous cell carcinoma cells

**DOI:** 10.1080/21655979.2021.1980644

**Published:** 2021-10-06

**Authors:** Jiahua Xing, Ziqi Jia, Yichi Xu, Muzi Chen, Zheng Yang, Youbai Chen, Yan Han

**Affiliations:** a Department of Plastic and Reconstructive Surgery, The First Medical Center, Chinese Pla General Hospital, Beijing, China; b School of Medicine, Nankai University, Tianjin, China; c Peking Union Medical College, Beijing, China

**Keywords:** KLF9, pfkfb3, proliferation, aerobic glycolysis, cutaneous squamous cell carcinoma cells

## Abstract

Cutaneous squamous cell carcinoma (CSCC) is the second most common skin cancer in humans with increasing incidence. In this paper, we focused on the effects of krueppel-like factor 9 (KLF9) on the progression of CSCC cells by binding to PFKFB3. mRNA and protein expressions of KLF9 and PFKFB3 in human HaCaT and CSCC cells were, respectively, examined by RT-qPCR analysis and Western blot. The viability, proliferation, invasion and migration of A431 cells after transfection were analyzed with MTT, clone formation, transwell and wound healing assays. The levels of glucose, lactic acid and ATP in transfected A431 cells were detected by their commercial kits. Ki-67 expression in transfected A431 cells was determined using immunofluorescence analysis and in tumor tissues was analyzed by immunohistochemistry. The levels of migration, EMT and aerobic glycolysis-related proteins were tested with Western blot. The combination of KLF9 and PFKFB3 was confirmed by dual-luciferase reporter assay and ChIP. As a result, PFKFB3 expression was elevated in CSCC cells compared with HaCaT. Knockdown of PFKFB3 restrained the proliferation, metastasis, and aerobic glycolysis of CSCC cells. In addition, KLF9 could bind to PFKFB3. Downregulation of KLF9 crippled the inhibitory effect of knockdown of PFKFB3 on the proliferation, metastasis, and aerobic glycolysis of CSCC cells. In conclusion, PFKFB3 was transcriptionally regulated by KLF9, and PFKFB3 silencing inhibits the proliferation, metastasis, and aerobic glycolysis of cutaneous squamous cell carcinoma cells.

## Introduction

Cutaneous squamous cell carcinoma (CSCC), whose incidence is continuing to increase but still second only to basal cell carcinoma in recent years, is a common skin cancer in humans with invasion and metastasis capabilities [1]. It often occurs on the basis of some skin diseases, including burns, scarring, discoid lupus erythematosus, hypertrophic lichen planus [2–5]. Among the existing etiological factors that induce the occurrence and development of CSCC, chronic ultraviolet (UV) radiation exposure is regarded as the major cause of CSCC [6–9]. A large variety of therapeutic approaches can be opted for the treatment of CSCC, and surgical resection is the optimal choice for CSCC, which is implicated in a large region of the patients’ skin [10]. Due to the strong invasion and metastasis abilities of CSCC, some patients still miss the best diagnosis and therapy time. Hence, it is still necessary to further study its pathogenesis and find new and more effective therapeutic methods.

6-phosphofructo-2-kinase/fructose-2,6-biphosphatase 3 (PFKFB3), a bifunctional glycolytic enzyme with two homodimer subunits that own two functional domains and, respectively, play the dual functions of kinase and phosphatase, respectively [11,12], can accelerate the process of glycolysis to provide energy and opportunities for the tumor cells to reproduce and invade. At present, a previous study has demonstrated that PFKFB3 is highly expressed in a large number of malignant tumor cells [13], such as prostate cancer, bowel cancer, gastric cancer, and pancreatic cancer, etc., and the enhanced level of this protein is associated with the poor prognosis of these diseases [14,15]. A study has shown that miR-3666 suppresses the growth of head and neck squamous cell carcinoma cells by inhibiting the Warburg effect mediated by PFKFB3 [16]. LncRNA AGPG is also highly expressed in many cancers and is closely related to poor prognosis. AGPG binds to and stabilizes PFKFB3, the accumulation of which in esophageal squamous cell carcinoma cells can thus activate glycolytic flux and promote cell cycle progression [17]. PFKFB3 expression in oral squamous cell carcinoma is notably increased and correlated with the differentiation degree and tumor size. PFKFB3 may promote tumor progression and angiogenesis during metastasis by regulating the infiltration of CD163+ TAMs in oral squamous cell carcinoma [18]. Inhibition of glycolysis by targeting PFKFB3 inhibits the growth and metastasis of squamous cell carcinoma of the head and neck [19]. However, the role of PFKFB3 in cutaneous squamous cell carcinoma has not been reported.

JASPAR (http://jaspar.genereg.net/) predicts that the transcription factor krueppel-like factor 9 (KLF9) binds to the PFKFB3 promoter. The human KLF9 gene, also known as BTEB, is located on chromosome 9ql3 and encodes a protein consisting of 144 amino acid residues [20]. A previous study also noted decreased KLF9 expression in esophageal squamous cell carcinoma (ESCC), and suggested the notion that KLF9 inhibited the growth and metastasis of ESCC cells [21]. MiR-652 negatively regulated KLF9 expression to enhance the proliferation and invasion of osteosarcoma [22]. KLF9 is obviously downregulated in patients with gastric cancer (GC) and KLF9 significantly inhibits the invasion and metastasis of GC cells through inhibiting the MMP28 transcription [23]. However, the function of KLF9 in CSCC remains undefined.

In the present study, PFKFB3 and KLF9 expression in several human CSCC cell lines were investigated. And the subsequent experiments focused on the effects of PFKFB3 on proliferation, metastasis and aerobic glycolysis of CSCC and whether it can be transcriptionally regulated by KLF9. Our findings may be a promising therapeutic strategy for CSCC.

## Material and methods

### The mouse A431 cell tumor xenograft model

Animal experiments were approved by the Ethics Committee of Chinese PLA General Hospital. Male nude mice (4–5 weeks, 14–16 g) were randomly assigned to small hairpin RNA (shRNA)-NC group and shRNA-PFKFB3 group (*n* = 3 per group). A total of 2 × 10^6^ A431 cells after indicated transfection in 100 μL of DMEM without FBS were injected into right axillary fossa of nude mice. The body weight of mice was measured using electronic scale every 3 days. Finally, the mice were euthanized with pentobarbital sodium (150 mg/kg, i.p.) on day 21 after tumor implantation to isolate the CSCC tumors, and the volume was measured using calipers every 3 days with the formula of (length × width^^2^)/2.

### Cell culture

Human immortalized keratinocytes (HaCaT) and human CSCC cells (SCL-1, SCC13, HSC-5 and A431) were purchased from BioVector NTCC Inc. (Beijing, China). Cells were incubated in Dulbecco’s modified Eagle’s medium (DMEM; Gibco, Grand Island, USA) added with 10% fetal bovine serum (FBS; Gibco, Grand Island, USA) at 37°C with 5% CO_2_.

### Cell transfection

ShRNA-negative control (NC), shRNA-PFKFB3#1, shRNA-PFKFB3#2, shRNA-KLF9#1, shRNA-KLF9#2, pcDNA3.1 and pcDNA3.1-KLF9 were constructed by GenePharma (Shanghai, China). A431 cells were transfected with the above plasmids by means of Lipofectamine 2000 reagent (Thermo Fisher Scientific) as provided by the manufacturer and incubated for another 48 h.

### Western blot analysis

HaCaT, human CSCC cells and transfected A431 cells were collected and lysed with ice-cold RIPA lysis buffer to obtain the proteins which were determined by BCA kit. A total of 30 μg protein were separated by 10% sodium dodecyl sulfate (SDS)-polyacrylamide gels and transferred onto a PVDF membrane. After blocking with 5% nonfat milk, these blots were incubated against PFKFB3, matrix metalloproteinase-2 (MMP-2), MMP-9, E-cadherin, N-cadherin, Vimentin, hypoxia-inducible-factor 1A (HIF1A), lactate dehydrogenase A (LDHA), glucose transporter type 1 (GLUT1), HK2, KLF9, and GAPDH, and then incubated with the HRP-conjugated secondary antibody for 1 h. The protein bands were visualized with the enhanced chemiluminescence reagents, of which the gray level was obtained by ImageJ software using GAPDH as the endogenous reference.

### RT-qPCR analysis

HaCaT, human CSCC cells and transfected A431 cells were collected and TRIzol® reagent was employed to extract the total RNA from above cells. Total RNA was reverse transcribed into complementary DNA (cDNA) using a PrimeScript RT Reagent kit (Takara Bio, Inc.) and qPCR was subsequently conducted with SYBR Green PCR Master Mix (Applied Biosystems) on an ABI Prism 7500 Real-Time PCR system. The primer sequences were as follows: PFKFB3 forward, 5′-CCTCACTCGCAGCCACTTCT-3′ and reverse, 5′-CAGTTCCTACTCAATTCCAA-3′; KLF9 forward, 5′-ACAGTGGCTGTGGGAAAGTC-3′ and reverse, 5′ – TCACAAAGCGTTGGCCAGCG-3′; and GAPDH forward, 5′-GGAGCGACATCCGTCCAAAAT-3′ and reverse, 5′-GGCTGTTGTCAATCTTCTCATGG-3′. mRNA expression levels of PFKFB3 and KLF9 were quantified by means of the 2^−ΔΔCq^ method [24].

### Methyl Thiazolyl Tetrazolium (MTT) assay

A431 cells with or without the indicated transfection were inoculated on 96-well plates and then cultured normally at 37°C with 5% CO_2_. At 24, 48 and 72 h, cells were added with 10 μl of MTT (Sigma, USA) for incubation of 4 h in the dark, and then added with 100 μl of dimethyl sulfoxide (DMSO; Sigma, USA) on a shaker for incubation of 10 min in dark. The optical density was measured at 490 nm by a microplate reader.

### Clone formation assay

A431 cells (1 × 10^3^ cells/well) with or without the indicated transfection were inoculated onto a 6-well plate and then cultured normally at 37°C with 5% CO_2_. After incubation for 7 days, the number of colonies was counted and photographed by a digital camera.

### Immunofluorescence analysis

A431 cells with or without the indicated transfection were climbing to the carry sheet glass, which was then fixed in ice-cold 4% paraformaldehyde and soaked in 0.5% Triton X-100 for 20 min at room temperature. The, cells were blocked with 3% bovine serum albumin (BSA) for 30 min and incubated with the anti-Ki-67 antibody. On the next day, followed by the PBS washing, cells were incubated with the secondary antibody conjugated with fluorescein isothiocyanate (FITC) for 1 h. Cell nuclei were counterstained with DAPI for 5 min in the dark. The Ki-67 expression was viewed under a fluorescence microscope.

### Immunohistochemistry

Paraffin-embedded CSCC tissues of mice were completely deparaffinized and added with antigen retrieval solution (Beyotime) for antigen retrieval. According to the manufacturer’s recommendation of Immunohistochemistry Application Solutions Kit (Cell Signaling Technology), the slides were incubated against Ki-67 and then incubated against the secondary antibody for 30 min. Finally, the slides were stained with 3, 3ʹ-diaminobenzidine (DAB, ZSGB-BIO) and counterstained with hematoxylin for 3 min, which were then photographed by a light microscope equipped with digital camera.

### Wound healing assay

A431 cells were treated with or without the indicated transfection and incubated normally to grow into full confluency in 6-well plates. Then, A431 cells were incubated overnight in the starvation medium. A sterile 100 μl pipette tip was utilized to scratch the cell monolayers, followed by the washing of starvation medium. Left A431 cells were incubated in full medium in a cell culture incubator for 48 h and the wound gap was photographed by a light microscope.

### Transwell assay

A431 cells (1 × 10^5^ cells/well) with or without the indicated transfection were added to the upper chamber precoated with matrigel, and the lower chamber was added with 500 μl of DMEM supplemented with 10% FBS. The plate was incubated for 48 h at 37°C. A431 cells were fixed with 4% paraformaldehyde, stained with crystal violet and photographed.

### Detection of glucose, lactic acid, and ATP

A431 cells with or without the indicated transfection were cultured normally for 24 h to collect the supernatant. The levels of glucose, lactic acid and ATP in the supernatant were analyzed with Glucose Assay kit (cat. no. E-BC-K234-S; Elabscience), L-Lactic Acid Colorimetric Assay kit (cat. no. E-BC-K044-S; Elabscience) and ATP Assay Kit (cat. no. S0026; Beyotime).

### Dual luciferase reporter assay

Luciferase reporter plasmids (Promega Corporation) were constructed with wild-type (WT) and mutant-type (MUT) 3ʹ-untranslated regions of PFKFB3 promoter by GenePharma (Shanghai, China). The luciferase reporter plasmids and KLF9-expressing plasmid were co-transfected into A431 cells with Lipofectamine 2000 (Thermo Fisher Scientific, Inc.). After 48 h of transfection, the Renilla and firefly luciferase activities were detected with the dual-luciferase reporter assay system (Promega Corporation). The data were normalized to Renilla luciferase.

### Chromatin Immunoprecipitation (ChIP)

A431 cells were cross-linked with 1% formaldehyde. After lysis and sonication, the supernatant was obtained by centrifugation. Samples were incubated with anti-KLF9 or control IgG in the presence of protein A/G beads. The enrichment of indicated proteins in PFKFB3 promoter was evaluated by PCR.

### Statistical analysis

Data were performed with the GraphPad Prism version 8 software. Quantitative data were presented as mean ± standard deviation (SD) from replicate experiments. Comparison among two groups was performed by using Student’s t-test. One-away analysis of variance (ANOVA) with Tukey’s post hoc test was employed to analyze significance among multiple groups. P < 0.05 suggested the statistical significance of difference.

## Results

### PFKFB3 is highly expressed in CSCC cells

**Figure 1.**
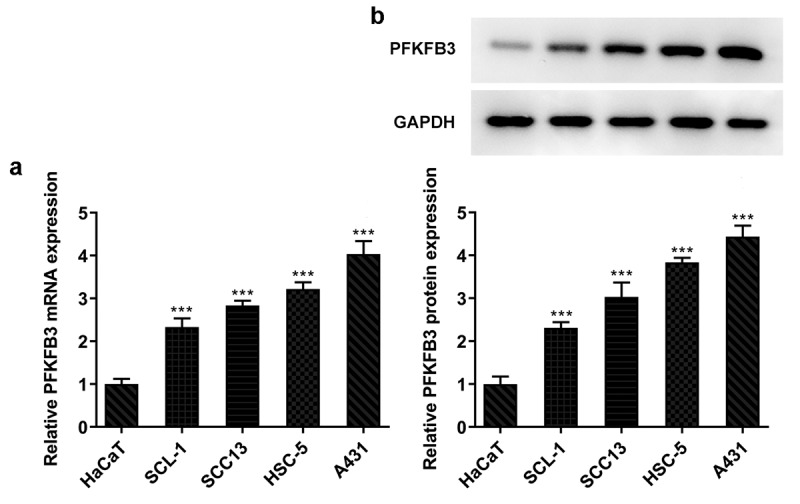
PFKFB3 is highly expressed in CSCC cells. (a) PFKFB3 mRNA expression and (b) PFKFB3 expression in CSCC cells and HaCaT cells was respectively examined using RT-qPCR and Western blot. ***P < 0.001 vs. HaCaT

Research has proposed that PFKFB3 is highly expressed in a large number of malignant tumor cells [13]. Therefore, the expression of PFKFB3 in several human CSCC cell lines was evaluated firstly. As shown in Figure 1a, PFKFB3 mRNA level was obviously elevated in CSCC cells when compared to that in HaCaT cells, and PFKFB3 mRNA level in A431 cells was the highest among CSCC cells. Similarly, PFKFB3 protein level was also remarkably enhanced in CSCC cells relative to that in HaCaT cells, and PFKFB3 protein level in A431 cells was also the highest among CSCC cells (Figure 1b). Therefore, A431 cell line was chosen for the subsequent experiments.

## PFKFB3 silencing suppresses the proliferation of CSCC cells

**Figure 2.**
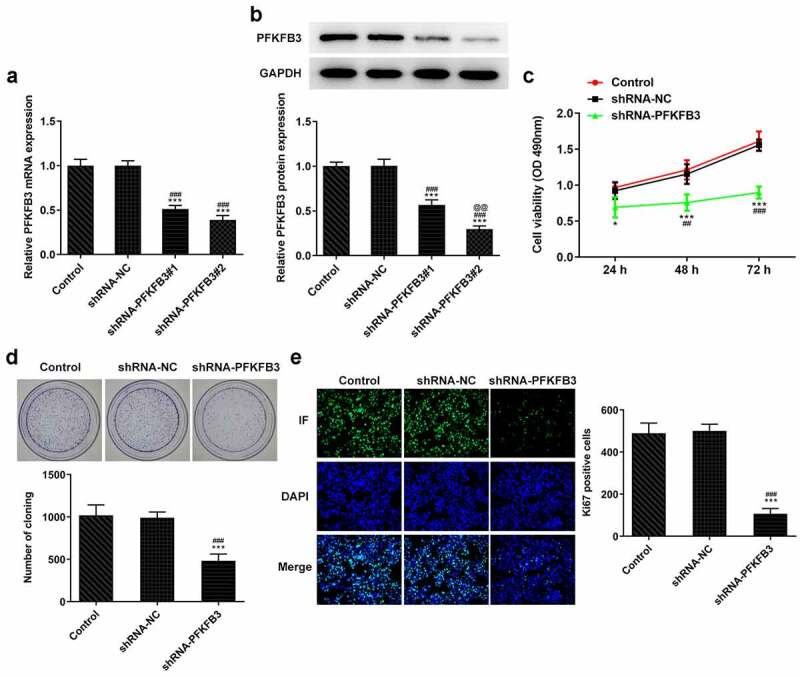
PFKFB3 silencing suppresses the proliferation of CSCC cells. (a) PFKFB3 mRNA expression and (b) PFKFB3 protein expression in A431 cells after transfection with shRNA-PFKFB3#1/2 was respectively tested by RT-qPCR and Western blot. (c) The viability, (d) proliferation and (e) Ki-67 expression of A431 cells transfected with shRNA-PFKFB3#2 was in turn analyzed by MTT assay, clone formation assay and immunofluorescence assay. *P < 0.05 and ***P < 0.001 vs. Control. ^##^P < 0.01 and ^###^P < 0.001 vs. shRNA-NC. ^@@^P < 0.01 vs. shRNA-PFKFB3#1

Then, the effects of PFKFB3on the progression of CSCC were explored by transfection with shRNA-PFKFB3#1/2, and the expression of PFKFB3 was determined with RT-qPCR and western blot assay. As presented in Figure 2a, PFKFB3 mRNA level was obviously decreased in A431 cells transfected with shRNA-PFKFB3#1/2. PFKFB3 protein expression was also significantly decreased in A431 cells transfected with shRNA-PFKFB3#1/2 and was lower in shRNA-PFKFB3#2 group than that in shRNA-PFKFB3#1 group (Figure 2b). Hence, shRNA-PFKFB3#2, which was generally expressed as shRNA-PFKFB3 in later, was selected to carry out the following experiments. In addition, PFKFB3 knockdown restrained the viability and proliferation of A431 cells (Figure 2c and 2b) and suppressed the Ki-67 expression (Figure 2e).

## PFKFB3 knockdown inhibits the metastasis of CSCC cells

**Figure 3.**
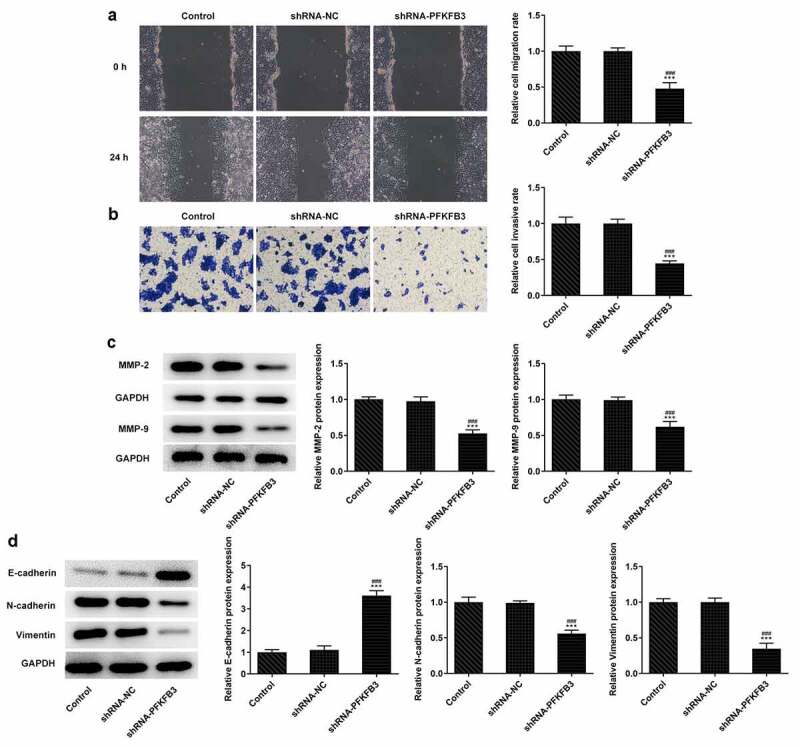
PFKFB3 knockdown restrains the metastasis of CSCC cells. (a) The migration and (b) invasion of A431 cells transfected with shRNA-PFKFB3#2 were respectively evaluated with wound healing assay and transwell assay. The levels of (c) migration and invasion related proteins and (d) EMT related proteins in A431 cells transfected with shRNA-PFKFB3#2 were assessed using Western blot. ***P < 0.001 vs. Control. ^###^P < 0.001 vs. shRNA-NC

The metastasis ability of CSCC lead to the poor prognosis, and invasion and migration are considered two important feature of it. Thus, the effects of PFKFB3 knockdown on invasion and migration of CSCC cells were explored. It has been found that PFKFB3 knockdown attenuated the migration and invasion of A431 cells (Figure 3a and 3b) and suppressed the expression of MMP-2 and MMP-9 (Figure 3c). For epithelial-mesenchymal transition (EMT), the E-cadherin level was markedly enhanced and levels of N-cadherin and Vimentin were markedly decreased in shRNA-PFKFB3 transfected A431 cells (Figure 3d).

## PFKFB3 knockdown inhibits the aerobic glycolysis of CSCC cells

**Figure 4.**
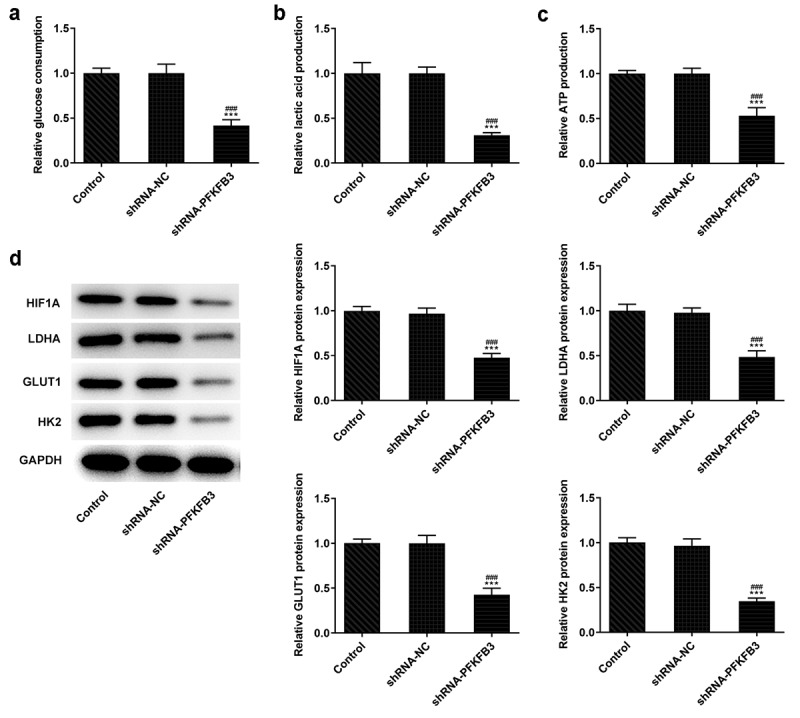
PFKFB3 knockdown inhibits the aerobic glycolysis of CSCC cells. (a) The glucose consumption, (b) lactic acid production and (c) ATP production in A431 cells transfected with shRNA-PFKFB3#2 were in turn detected by Glucose Assay kit, L-Lactic Acid Colorimetric Assay kit and ATP Assay Kit. (d) The levels of aerobic glycolysis related proteins in A431 cells transfected with shRNA-PFKFB3#2 was analyzed by Western blot. ***P < 0.001 vs. Control. ^###^P < 0.001 vs. shRNA-NC

During the development of CSCC, glycolysis can provide energy and opportunities for the tumor cells to reproduce and invade [25]. Whether PFKFB3 downregulation affecting glycolysis has been analyzed in the following experiments. We found that PFKFB3 knockdown obviously decreased the glucose consumption (Figure 4a), lactic acid production (Figure 4b) and ATP production (Figure 4c). The expression of aerobic glycolysis-related proteins (HIF1A, LDHA, GLUT1, and HK2) was also significantly downregulated (Figure 4d).

## KLF9 negatively modulated PFKFB3 transcription in CSCC

**Figure 5.**
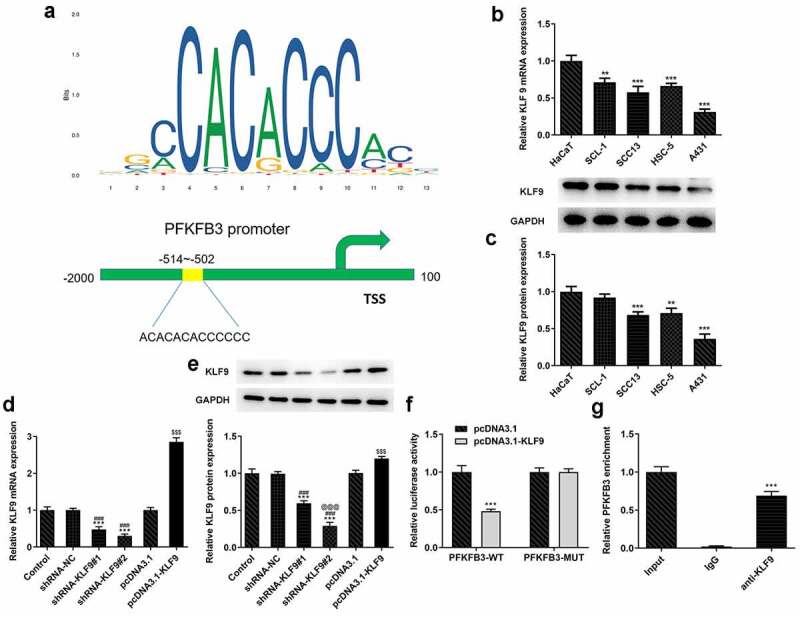
KLF9 negatively modulated PFKFB3 transcription in CSCC. (a) The binding sites between KLF9 and PFKFB3. (b) KLF9 mRNA expression and (c) KLF9 protein expression in several CSCC cells and HaCaT cells was respectively determined with RT-qPCR and Western blot. **P < 0.01 and ***P < 0.001 vs. HaCaT. (d) KLF9 mRNA expression and (e) KLF9 protein expression in A431 cells after transfection was measured with RT-qPCR and Western blot. ***P < 0.001 vs. Control. ^###^P < 0.001 vs. shRNA-NC. ^@@@^P < 0.001 vs. shRNA-KLF9#1. ^$$$^P < 0.001 vs. pcDNA3.1. (f)The interaction between KLF9 and PFKFB3 was analyzed by dual-luciferase reporter assay. ***P < 0.001 vs. pcDNA3.1. (g) The binding ability of KLF9 to PFKFB3 promoter was evaluated with ChIP. ***P < 0.001 vs. IgG

JASPAR predicted the binding sits between KLF9 and PFKFB3 (Figure 5a). KLF9 mRNA and protein expressions were significantly decreased in CSCC cells when compared to the HaCaT cells, and the lowest KLF9 expression was observed in A431 cells (Figure 5b and 5c). KLF9 mRNA expression and protein expressions were dramatically reduced in shRNA-KLF9#1/2 group and increased in pcDNA3.1-KLF9 group. KLF9 protein expression in shRNA-KLF9#2 group was lower than that in shRNA-KLF9#1 group (Figure 5d and 5e). Therefore, shRNA-KLF9#2, which was generally expressed as shRNA-KLF9 in later, was used to conduct the subsequent experiment. The relative luciferase activity in A431 cells co-transfected with PFKFB3-WT and pcDNA3.1-KLF9 was decreased relative to that co-transfected with PFKFB3-MUT and pcDNA3.1-KLF9 (figure 5f). The result of ChIP demonstrated that KLF9 combined with PFKFB3 (Figure 5g).

**KLF9 knockdown restored the impact of PFKFB3 knockdown on the proliferation, metastasis, and aerobic glycolysis of CSCC cells.**

**Figure 6.**
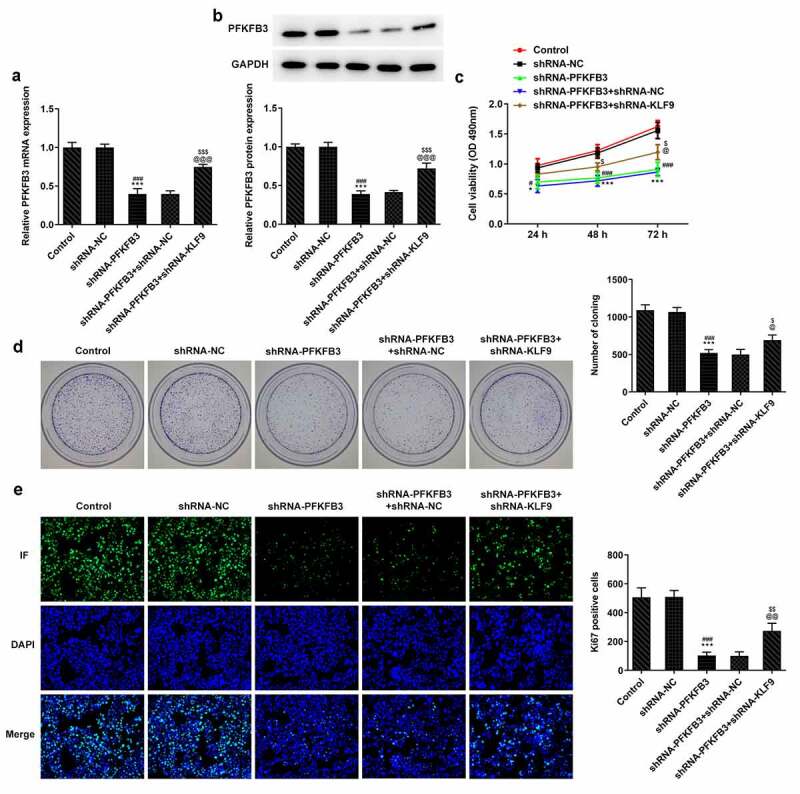
KLF9 knockdown attenuated the impacts of PFKFB3 knockdown on the proliferation of CSCC cells. (a) PFKFB3 mRNA expression and (b) PFKFB3 protein expression in A431 cells transfected with shRNA-PFKFB3#2 and shRNA-KLF9#2 was respectively detected using RT-qPCR and Western blot. (c) The viability, (d) proliferation and (e) Ki-67 expression of A431 cells transfected with shRNA-PFKFB3#2 and shRNA-KLF9#2 was tested with MTT assay, clone formation assay and immunofluorescence analysis. *P < 0.05 and ***P < 0.001 vs. Control. ^#^P < 0.05 and ^###^P < 0.001 vs. shRNA-NC. ^@^P < 0.05 and ^@@@^P < 0.001 vs. shRNA-PFKFB3. ^$^P < 0.05 and ^$$$^P < 0.001 vs. shRNA-PFKFB3 + shRNA-NC

**Figure 7.**
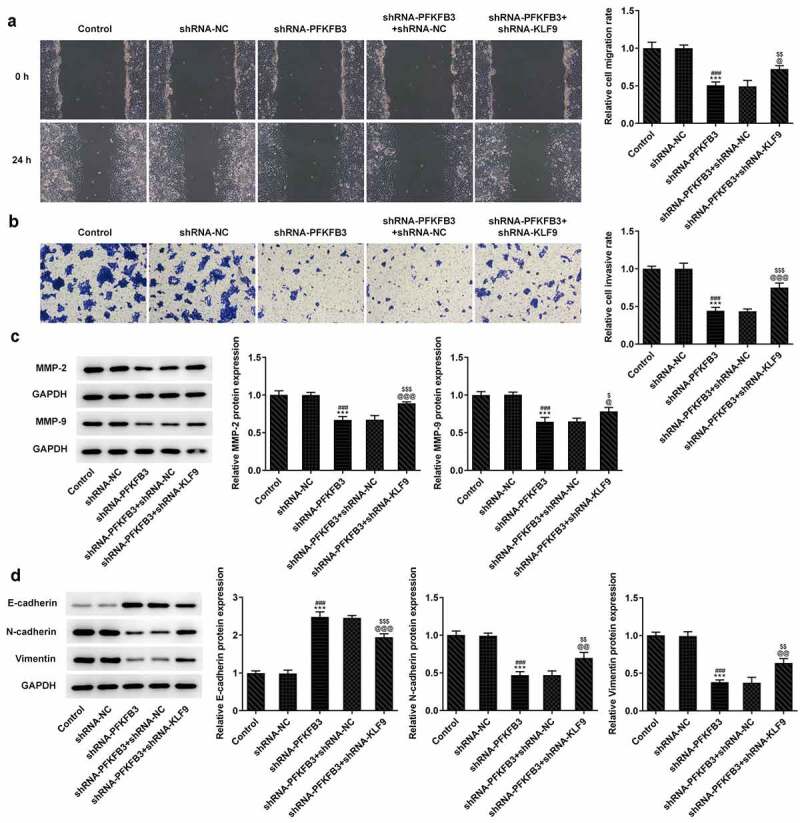
KLF9 knockdown reversed the inhibitory effect of PFKFB3 knockdown on the metastasis of CSCC cells. (a) The migration and (b) invasion of A431 cells transfected with shRNA-PFKFB3#2 and shRNA-KLF9#2 were assessed with wound healing assay and transwell assays. The expression of (c) migration and invasion related proteins and (d) EMT related proteins in A431 cells transfected with shRNA-PFKFB3#2 and shRNA-KLF9#2 was examined by Western blot. ***P < 0.001 vs. Control. ^###^P < 0.001 vs. shRNA-NC. ^@^P < 0.05, ^@@^P < 0.01 and ^@@@^P < 0.001 vs. shRNA-PFKFB3. ^$^P < 0.05, ^$$^P < 0.01 and ^$$$^P < 0.001 vs. shRNA-PFKFB3 + shRNA-NC

**Figure 8.**
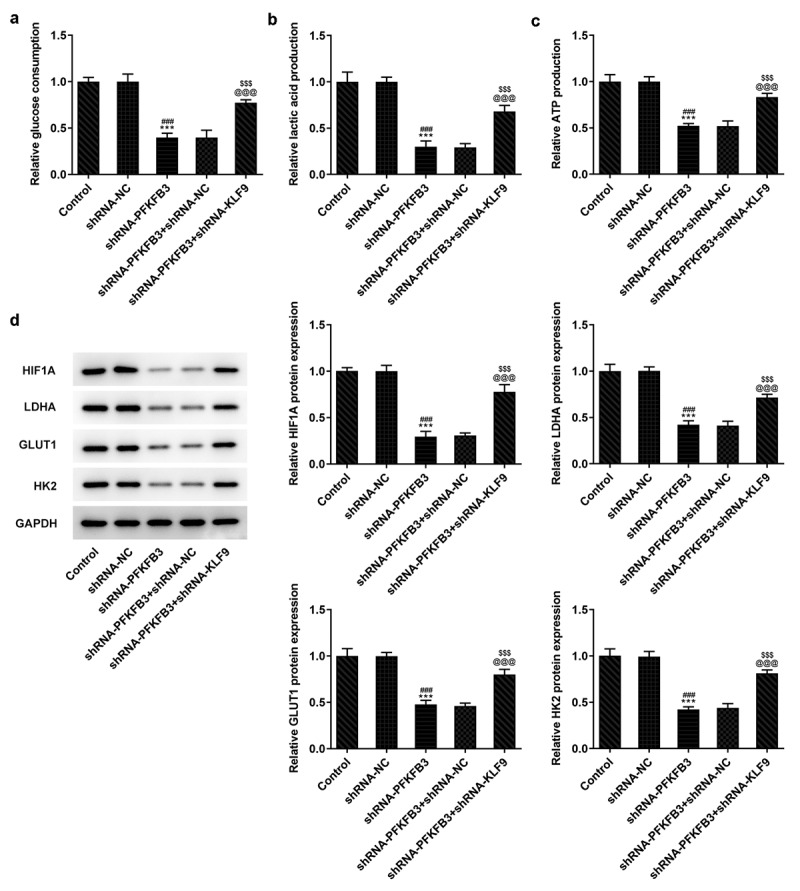
KLF9 silencing reversed the inhibitory effect of PFKFB3 knockdown on the aerobic glycolysis of CSCC cells. (a) The glucose consumption, (b) lactic acid production and (c) ATP production in A431 cells transfected with shRNA-PFKFB3#2 and shRNA-KLF9#2 were examined using Glucose Assay kit, L-Lactic Acid Colorimetric Assay kit and ATP Assay Kit. (d) The levels of aerobic glycolysis related proteins in A431 cells transfected with shRNA-PFKFB3#2 and shRNA-KLF9#2 was tested with Western blot. ***P < 0.001 vs. Control. ^###^P < 0.001 vs. shRNA-NC. ^@@@^P < 0.001 vs. shRNA-PFKFB3. ^$$$^P < 0.001 vs. shRNA-PFKFB3 + shRNA-NC

To further clarify whether the effects of PFKFB3 knockdown on the proliferation, metastasis, and aerobic glycolysis of CSCC cells were functioned by being transcriptional activation by KLF9, KLF9 was silenced. As exhibited in Figure 6a and 6b, KLF9 knockdown obviously upregulated the PFKFB3 mRNA expression and PFKFB3 protein expression in shRNA-PFKFB3 transfected A431 cells. KLF9 knockdown also improved the viability and proliferation of shRNA-PFKFB3 transfected A431 cells (Figure 6c and 6d). The Ki-67 expression was also increased by KLF9 knockdown in shRNA-PFKFB3 transfected A431 cells (Figure 6e). KLF9 knockdown also improved the migration and invasion of shRNA-PFKFB3 transfected A431 cells (Figure 7a and 7b), and MMP-2 and MMP-9 levels in shRNA-PFKFB3 transfected A431 cells were also upregulated by KLF9 knockdown (Figure 7c). KLF9 knockdown could downregulate the E-cadherin level and upregulate N-cadherin and Vimentin levels in shRNA-PFKFB3 transfected A431 cells (Figure 7d). The glucose consumption (Figure 8a), lactic acid production (Figure 8b) and ATP production (Figure 8c) were all improved by KLF9 knockdown in shRNA-PFKFB3 transfected A431 cells, and the aerobic glycolysis-related protein expression was also upregulated (Figure 8d).

## PFKFB3 knockdown inhibited the growth of CSCC in vivo

**Figure 9.**
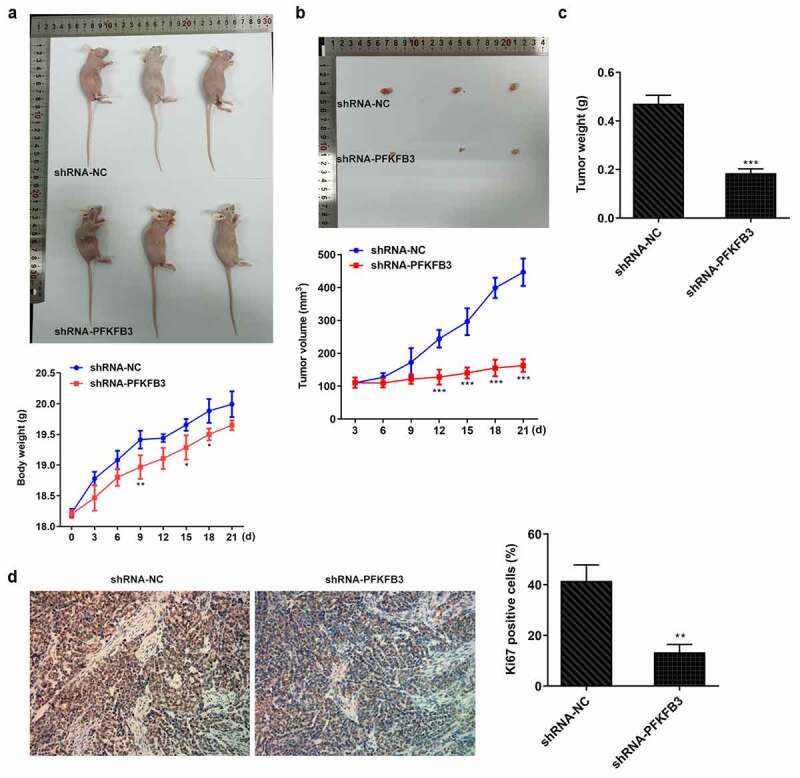
PFKFB3 knockdown by transfection with shRNA-PFKFB3#2 inhibited the growth and metastasis of CSCC in vivo. (a) The growing curve of mice body weight. (b) The growing curve of mice tumor volume. (c) Mice tumor weight. (d) Ki-67 expression in tumor tissues was measured with immunohistochemistry. Magnification, x200. *P < 0.05, **P < 0.01 and ***P < 0.001 vs. shRNA-NC

Subsequently, the effects of PFKFB3 knockdown on the growth of CSCC in vivo were investigated. The body weight of mice was not obviously changed between shRNA-NC group and shRNA-PFKFB3 group (Figure 9a). From day 12 to day 21, tumor volume was obviously decreased (Figure 9b), and tumor weight was also decreased at day 21 (Figure 9c) in shRNA-PFKFB3 group as comparison to that in shRNA-NC group. In addition, Ki-67 expression was decreased in tumor tissues of shRNA-PFKFB3 group relative to that in shRNA-NC group.

## Discussion

Transcription factor is a kind of protein in the nucleus that can bind to DNA specifically and modulate the expression of target genes. It exerts significant effects on the physiological and biochemical processes, such as cell proliferation, growth, differentiation, and apoptosis [26]. In tumors, transcription factors usually present different expression levels in normal cells and participate in the development of human cancers [27]. In endometrial cancer cell line Hec-1-A, the stability of KLF9 expression could increase DNA synthesis and cell cycle dynamics by inducing cell cycle-related genes [28]. Compared with normal mucosal cells, the abnormally low KLF9 level in colorectal cancer tissues was correlated with tumor stage [29]. KLF9 could regulate the growth of tumor stem cells through the Notch1 signaling pathway, induce the differentiation of neural bulb cells and suppress the growth of tumor stem cells in glioma extended neural bulb cells, and it was low expressed in clinical glioma tissue samples [29]. In non-small-cell lung cancer, KLF9 expression was downregulated in tumor tissues and cells and KLF9 could suppress the promotion effect of miR-141 on the progression on A549 cells [30]. KLF9 expression was suppressed by miR-636 to enhance the proliferation of bladder cancer cells [31]. Here, we demonstrated that KLF9 expression was also decreased in A431 cells and KLF9 knockdown could promote the proliferation, metastasis, and aerobic glycolysis of A431 cells.

The transcription factor KLF9 has been demonstrated to combine with the PFKFB3 promoter. The expression of PFKFB3 is the highest in human tumor cells in situ, and its enzyme activity, which is about 700 times of phosphatase, is conducive to the synthesis of F-2 and 6-BP, the most potent stimulants for glycolysis [13]. Meanwhile, clear evidence shows that PFKFB3, when used as a key enzyme of glycolysis, is also notably elevated in liver cancer [32], osteoma [33], breast cancer [34] and NSCLC [35], indicating the tight association of PFKFB3 with the prognosis of tumors. Abnormal glycolytic pathway is a very important characteristic of malignant tumors, which confers tumors specific colonization ability [36]. Upon activation, the glycolytic pathway will facilitate the growth of gastric cancer. During the development of CSCC, glycolysis can provide energy and opportunities for the tumor cells to reproduce and invade [25]. Additionally, the enhancement of glucose metabolism in tumor cells is related to the activity of rate-limiting enzymes during glycolysis [37,38]. Here, PFKFB3 expression was also upregulated in CSCC cells, and PFKFB3 knockdown could suppress the aerobic glycolysis of A431 cells, thereby inhibiting the cell proliferation and impeding the development of CSCC.

It is widely accepted that the metastasis of tumor cells is associated with the degradation of extracellular matrix (ECM) as the ECM degradation enzymes synthesized by tumor cells can accelerate the invasion and migration of cells [39]. MMP-2 and MMP-9 are members of the matrix metalloproteinase family that can decompose ECM and induce tumor metastasis [40,41]. EMT is a key cellular transformation process in malignant tumors, which can lead to invasion and migration of a variety of tumor cells [42,43]. Biochemically, cells suppress the level of epithelial markers (E-cadherin) and enhance the levels of mesenchymal markers (vimentin and fibronectin) [44,45]. In this study, PFKFB3 knockdown suppressed the invasion and migration of A431 cells by inhibiting MMP-2, MMP-9, N-cadherin and Vimentin expression while promoting the E-cadherin expression.

## Conclusion

Taken together, KLF9-induced PFKFB3 downregulation inhibits the proliferation, metastasis and aerobic glycolysis of cutaneous squamous cell carcinoma cells. In addition, PFKFB3 knockdown could also suppress the tumor growth in vivo. The present finding may be a promising therapeutic strategy for CSCC.

## Data Availability

The experimental data will be available on the request.
